# A Smartphone-Based Intervention as an Adjunct to Standard-of-Care Treatment for Schizophrenia: Randomized Controlled Trial

**DOI:** 10.2196/29154

**Published:** 2022-03-28

**Authors:** S Nassir Ghaemi, Oleksandr Sverdlov, Joris van Dam, Timothy Campellone, Robert Gerwien

**Affiliations:** 1 Novaris Institutes for Biomedical Research Cambridge, MA United States; 2 Novartis Pharmaceuticals Corporation East Hanover, NJ United States; 3 Pear Therapeutics Boston, MA United States

**Keywords:** digital therapeutics, schizophrenia, smartphones, randomized controlled trial, mobile phone

## Abstract

**Background:**

Antipsychotic medications have limited benefits in schizophrenia, and cognitive behavioral therapy may be beneficial as an adjunct. There may be potential for implementing mobile cognitive behavioral therapy–based treatment for schizophrenia in addition to standard antipsychotic medications.

**Objective:**

This study aims to determine whether PEAR-004, a smartphone-based investigational digital therapeutic, improves the symptoms of an acute psychotic exacerbation of schizophrenia when it is added to standard treatments.

**Methods:**

This was a 12-week, multicenter, randomized, sham-controlled, rater-blinded, parallel group proof‑of‑concept study of 112 participants with moderate acute psychotic exacerbation in schizophrenia. This study was conducted in 6 clinical trial research sites in the United States from December 2018 to September 2019. The primary outcome, change in Positive and Negative Syndrome Scale (PANSS) from baseline to week 12 or the last available visit, was analyzed using the mixed-effects regression model for repeated measures, applied to an intent-to-treat sample.

**Results:**

The total PANSS scores slightly decreased from baseline over the study period in both groups; the treatment difference at day 85 between PEAR-004 and sham was 2.7 points, in favor of the sham (2-sided P=.09). The secondary scales found no benefit, except for transient improvement in depressive symptoms with PEAR-004. Application engagement was good, and patient and clinical investigator satisfaction was high. No safety concerns were observed. There was some evidence of study site heterogeneity for the onboarding processes and directions on PEAR-004 product use at baseline and throughout the study. However, these differences did not affect the efficacy results.

**Conclusions:**

In the largest-to-date randomized, sham-controlled study of a digital therapeutic in schizophrenia, PEAR-004 did not demonstrate an effect on the primary outcome—total PANSS scores—when compared with a nonspecific digital sham control. The secondary and exploratory results also did not demonstrate any notable benefits, except for possible temporary improvement in depressive symptoms. This study provided many useful scientific and operational insights that can be used in the further clinical development of PEAR-004 and other investigational digital therapeutics.

**Trial Registration:**

ClinicalTrials.gov NCT03751280; https://clinicaltrials.gov/ct2/show/NCT03751280

## Introduction

Schizophrenia is a common condition [1] treated with standard antipsychotic medications, which can help acute exacerbations of delusions or hallucinations but do not improve the long-term course of the illness. Cognitive behavioral therapy (CBT) has been shown to improve the symptoms of schizophrenia both for delusions and hallucinations (*positive* symptoms) and for apathy and flat affect (*negative* symptoms) and functional status (engagement with employment or social interactions) [2]. However, access to CBT can be difficult based on the cost and availability of psychotherapists [3].

Digital therapeutics (DTx) represents a novel treatment modality in which digital technology systems are used as evidence-based therapeutic interventions to prevent, manage, or treat a medical disorder or disease [4]. DTx products based on CBT can be delivered through smartphone apps, and they can potentially provide safe, inexpensive, easy-to-use, consistent quality, personalized treatment strategies for patients with medical needs. The potential value of DTx is magnified during the current COVID-19 pandemic, when face-to-face physician visits are problematic and self-management of long-term conditions is becoming more common. There is an increasing interest in developing DTx for mental health disorders [5,6]. However, the evidence base for the clinical effectiveness of such products is still sparse [7,8]. There are natural challenges in designing clinical trials of DTx interventions, including the choice of a control group, blinding, and potentially lower-than-expected patient engagement [9].

Several digital interventions in psychosis have recently been evaluated in randomized controlled trials (RCTs) [10-12]. The experimental treatments in these studies varied in terms of mode of delivery, features or functionalities, and theoretical framework. In addition, there were different control conditions and different degrees of involvement of health professionals. For instance, Actissist (University of Manchester) [10] was developed as a stand-alone self-management mobile app targeting five domains of early psychosis, such as auditory verbal hallucinations, paranoia, perceived criticism, socialization, and cannabis use. It was tested in a small (n=36) proof-of-concept RCT against an active control condition (ClinTouch), which was a self-reporting symptom severity mobile app. Although the study [10] showed evidence of feasibility, acceptability, safety, and indications of beneficial effects after 12 weeks of treatment with Actissist, its findings are limited owing to the small sample size and the fact that engagement with both experimental and control apps was incentivized.

Another mobile app intervention, Personalized Real-Time Intervention for Motivation Enhancement (PRIME) [11], was designed to target the motivational system of young people with recent-onset schizophrenia spectrum disorders by using social reinforcement to engage and sustain goal-directed behavior. The PRIME intervention provided a supportive web-based environment for social interaction, motivational coaching, personalized goal setting in the domains of health or wellness, social relationships, creativity, productivity, and a system to track progress in achieving personal goals. After 12 weeks of PRIME treatment, there was evidence of improvement in the experimental group of several important components of motivational behavior, depression symptoms, defeatist beliefs, and self-efficacy, compared with the waitlist control condition. Despite a relatively small sample size (a total of 43 participants) and the use of a waitlist control that did not allow for assessment of a relative effect of PRIME compared with other mobile treatment approaches, the study [11] provided important evidence of feasibility, acceptability, and potential clinical benefit of a mobile intervention in this patient population. Notably, this study was implemented fully remotely, across the United States, Canada, and Australia.

The Audio Visual Assisted Therapy Aid for Refractory auditory hallucinations (AVATAR) therapy [12] is a computer-assisted intervention designed on the principles of CBT for psychosis, with the specific aim of controlling persistent, distressing auditory verbal hallucinations. The AVATAR therapy is delivered by experienced clinicians and involves the creation of a computerized representation of the entity (Avatar), which is believed to be the source of the voice heard by the patient, and subsequent therapy sessions at which the clinician facilitates a direct dialogue between the patient and the Avatar, with the goal of having the Avatar less hostile and conceding power over the course of therapy. In the RCT [12], which was formally powered (150 patients randomized equally between the AVATAR therapy and the supportive counseling control condition), the AVATAR therapy led to significantly greater reductions in auditory hallucinations, as assessed by the Psychotic Symptoms Rating Scales Auditory Hallucinations total score, compared with the control condition after 12 weeks of treatment; however, there was no between-group difference at 24 weeks follow-up. The study [12] was conducted at a single center and involved experienced therapists, which limits the generalizability of the results to other centers or to delivery by a wider mental health workforce.

FOCUS is another software-based intervention (delivered via mobile devices) designed with input from both treatment providers and patients to optimize both usability and engagement and developed to be used in conjunction with ongoing outpatient treatment [13]. The feasibility, acceptability, and initial efficacy of FOCUS for improving symptoms and treatment engagement in patients with schizophrenia or schizoaffective disorder was established in a 1-month open-label trial [14]. In another study [15], engagement with FOCUS among patients with schizophrenia was measured during a 6-month period following psychiatric hospitalization discharge. Similar to findings from the 1-month feasibility trial, patients with schizophrenia were highly engaged over the course of 6 months (active use on 82% of the weeks during which they had access to the intervention). Taken together, these preliminary findings regarding FOCUS show the promise of prescription DTx for improving symptoms and treatment outcomes in patients with schizophrenia.

Overall, theory-based digital interventions hold promise in psychosis and schizophrenia spectrum disorders. Given the increasing availability and use of smartphones, CBT‑based mobile interventions may potentially augment existing standard-of-care pharmacological treatments or target specific domains of illness by promoting cognitive and behavioral change strategies.

PEAR-004 is being developed as a prescription digital therapeutic delivered via smartphone for schizophrenia patients who are under the care of a qualified health care professional and are on antipsychotic pharmacotherapy. It is intended to deliver multimodal evidence-based neurobehavioral mechanisms of action, which include cognitive restructuring, illness self-management training, and social skills training. If efficacious, it would demonstrate that 24×7 access to evidence-based coping skills, which when added to medications, may improve symptom management and functional outcomes.

This paper reports the results of a randomized, sham-controlled study of PEAR-004 in patients with schizophrenia.

## Methods

### Study Design

The study was a multicenter, randomized, sham-controlled, rater-blinded, parallel group proof‑of‑concept trial of participants with schizophrenia (trial registration: ClinicalTrials.gov NCT03751280). Eligible participants were equally randomized on day 1 to receive either PEAR-004 (investigational digital therapeutic) or sham (control) for a period of 12 weeks. Participants in both groups continued to receive clinician-directed standard-of-care for schizophrenia, including pharmacotherapy. The participants returned to the clinic for outpatient visits at week 4 (day 29), week 8 (day 57), and week 12 (day 85). At each visit, standard assessments, including efficacy and safety, were performed according to the assessment schedule. A final follow-up visit was performed at week 16 (day 115).

### Study Objectives

The primary objective was to compare the effect of PEAR-004 versus sham, as assessed by the change from baseline to day 85 in the total Positive and Negative Syndrome Scale (PANSS) score. It was hypothesized that the PEAR-004 group would exhibit a greater reduction in the total PANSS score than the sham group. In addition, retention to assigned study treatment (dropout rate), patient engagement data, secondary efficacy outcomes, clinician outcome assessments, and safety and tolerability data were analyzed.

### Study Sites

Potentially eligible participants were enrolled in 6 investigational study sites in the United States. The site details are as follows:

- 1001: Collaborative Neuroscience Network, Garden Grove, California. Principal investigator (PI): Dr David Walling.
- 1002: Collaborative Neuroscience Network, Torrance, California. PI: Dr Lara Shirikjian.
- 1003: Pacific Research Partners, Oakland, California. PI: Dr Corinna Gamez.
- 1005: Meridien Research, Maitland, Florida. PI: Dr Andrea Marraffino.
- 1006: Cherry Health, Grand Rapids, Michigan. PI: Dr Eric Achtyes.
- 1008: Albuquerque Neuroscience, Albuquerque, New Mexico. PI: Dr Glenn Dempsey.

### Study Participants

The investigators ensured that all participants being considered for the study met the eligibility criteria at screening. The inclusion and exclusion criteria are described in Textbox 1.

**Inclusion and exclusion criteria.**

**Inclusion criteria**

- Signed informed consent obtained before participation in the study
- Healthy men and women aged 18 to 65 years, inclusive, and in good health as determined by medical history, physical examination, and vital signs at screening
- Structured Clinical Interview for DSM-5–based Diagnostic and Statistical Manual of Mental Disorders, 5th edition (DSM-5) diagnosis of schizophrenia and a total Positive and Negative Syndrome Scale score ≥60.
- Proficient in English at the 5th grade reading level or higher, in the judgment of the investigator
- Capable of using a mobile device (compatible with PEAR-004) and using common apps, in the judgment of the investigator

**Exclusion criteria**

- Major change in primary antipsychotic medication in the prior 4 weeks before screening
- Planning to move out of the geographic area within 3 months
- Unable to use English to participate in the consent process, the interventions, or assessments
- Inability to comply with study procedures owing to severe medical conditions or otherwise
- Meet the DSM-5 diagnosis for a current episode of major depression, mania, or hypomania in the past month
- Meet the DSM-5 diagnosis for a current moderate or severe alcohol or cannabis use disorder in the past 2 months
- Meet the DSM-5 diagnosis for a current substance use disorder (other than alcohol or cannabis) in the past 2 months
- Considered high risk for suicidal behavior based on InterSePT Scale for Suicidal Thinking–Plus score at screening, or in the judgment of the investigator
- Previously participated in a clinical study involving PEAR-004

### Randomization

**Figure 1:**
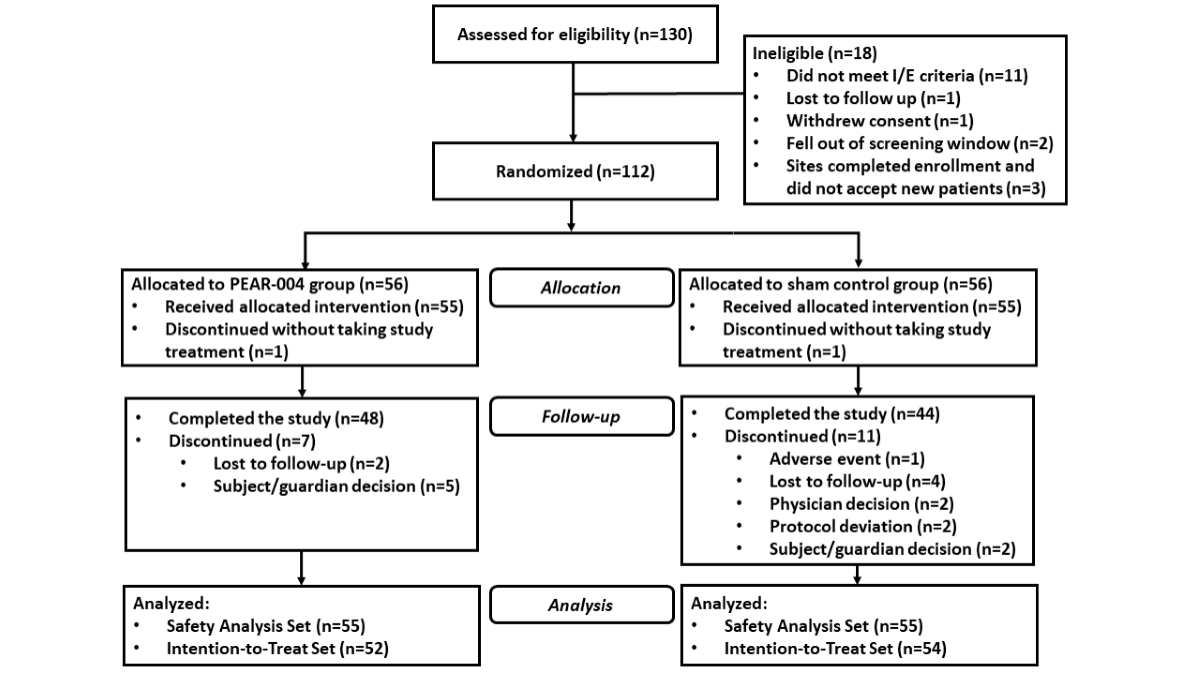
Study participant flowchart—CONSORT (Consolidated Standards of Reporting Trials) diagram. Safety analysis set: all subjects who received the study treatment. Intention-to-treat set: all subjects to whom study treatment has been assigned by randomization and who have a baseline observation and at least 1 postrandomization observation for the analysis end point. I/E: inclusion and exclusion.

Randomization was implemented by means of interactive response technology (IRT) using permuted blocks of size 4, with a targeted allocation ratio of 1:1. An investigator or delegate at a given study site logged on to the IRT system after confirming that the participant fulfilled all the inclusion or exclusion criteria. The IRT assigned the participant to a treatment arm, which was used by the site staff to request a *prescription access code* in a separate study portal. Trained site staff assisted the participants with all treatment onboarding activities. The participants were required to use their own personal mobile phones for the study. If a participant did not have their own phone, one was provided to them for use during the study. Additional information on the prescription access code and distribution of study treatment is provided in the site operational manuals provided to the sites. If a participant failed to be treated for any reason, the IRT was updated so that the participant was not treated. Treatment arm assignments were recorded in the case report form. Figure 1 shows the CONSORT (Consolidated Standards of Reporting Trials) participant flow through the trial.

### Interventions

On day 1, eligible participants gained access to either PEAR-004 or sham according to their randomization assignment. A single app containing both PEAR-004 and sham was downloaded from the iOS or Android app store to the participant’s mobile device, and then the assigned app was unlocked using a prescription access code provided by Pear Therapeutics. The study site staff received training on how to download PEAR-004 or sham to the assigned participant’s phone as part of site initiation activities. A single version of PEAR-004 or sham was used for the duration of the clinical study for all randomized participants.

The PEAR-004 smartphone app (iOS and Android-based) was designed as an illness self-management tool. Participant use of PEAR-004 during the treatment period could be either prompted or on demand. Prompted use refers to engagement initiated via 1 of the 3 daily notifications delivered at the following fixed times: 11 AM, 4 PM, and 9 PM. A participant responding to the notification would be brought to a unique survey asking whether the app can be helpful right now. Participants were free to choose from a list of modules or indicate that they were doing well and did not need help. If they chose a module, they would be brought directly to that module. If they indicated that they were doing well, they would be brought to the PEAR-004 home screen. In addition, PEAR-004 was available on demand. For a particular skill, there would be an option of watching, reading, or listening to content about what the skill is and how they can give it a try. Patients would then view tips for successful practice, and after they practiced a skill, they would be asked to provide feedback on whether the skill was helpful. Helpful skills were stored in the toolbox to promote repeated practice and skill mastery. The final PEAR-004 digital app used in this study was developed from prior versions to have 10 categories of skills: exercise, medication, mindfulness, mood, productivity, sleep, social activity, stress, thoughts, and voices. These categories were informed directly by user research (surveys and interviews) with people with schizophrenia.

In the sham control group, the sham app was downloaded to the participant’s phone but did not deliver the active therapeutic content of PEAR-004. Similar to PEAR-004, the sham app delivered 3 daily notifications prompting the participant to open the sham app, and then displayed a prescription timer (*digital clock*) for the remaining duration of app availability. The sham control arm was chosen to account for the nonspecific effects of engagement with a smartphone. The sham app did not deliver any active coping skills (ingredients of a psychosocial intervention). It appeared similar to the PEAR-004 app in its initial screen and randomization to sham or PEAR-004 was conducted at the time of app download unbeknownst to participants to maintain blinding.

Overall, the similarity and difference between the PEAR-004 app and the sham control app can be summarized as follows:

- Both PEAR-004 and sham sent 3 notifications per day at fixed times—11 AM, 4 PM, and 9 PM—prompting the user to open the app. Once the app was opened, the experience and content differed between the PEAR-004 and sham groups.
- PEAR-004 provided therapeutic content (cognitive and behavioral exercises), whereas the sham app did not.
- Sham had a display of a *digital clock*, whereas the PEAR-004 app did not.
- Neither PEAR-004 nor sham maintained a record of the user’s prescription medications or when they were to be taken.

### Assessments

#### Demographics and Baseline Characteristics

Participant demographic and baseline characteristic data were collected from all the study participants. Relevant medical history and current medical conditions present before signing informed consent were recorded.

#### Engagement With App

Data on patient engagement with the assigned app were collected throughout the study. The cross-platform engagement metrics (for individuals in both PEAR-004 and sham groups) included time using the app, number of days when the app was active, total number of sessions, and number of sessions per day. For participants in the PEAR-004 group, additional metrics were derived, such as the number of skills practiced, number of skills repeated (practiced at least two times), number of skills mastered (practiced at least three times), and number of skills practiced in each of the 10 categories of the PEAR-004 app (exercise, medication, mindfulness, mood, productive, sleep, social, stress, thoughts, and voices).

#### Efficacy

The following assessments were performed at each study visit:

- PANSS [16]: a 30-item clinician-administered, semistructured interview of schizophrenia symptoms. The PANSS assesses positive (hallucinations, delusions, and thought disorder) and negative (blunted affect, abstract thinking, and general symptomatology). The positive and negative subscale each consist of 7 items rated from 1 (absent) to 7 (extreme) with a minimum score of 7 and maximum score of 49. The general subscale consists of 16 items with a minimum score of 16 and a maximum score of 112. The total PANSS score (positive+negative+general scores) has a minimum of 30 and a maximum of 210. Higher scores represent greater symptom severity.
- Motivation and Pleasure-Self Report (MAP-SR) [17]: a 15-item self-report that provides a total score index of current motivation or pleasure negative symptoms. MAP-SR includes questions about social pleasure, recreational or work pleasure, close relationships, and motivation and effort to engage in activities of 15 questions with a score of 0-4, summed for a total range of 0-60. Higher values represent better outcomes.
- The Beck Depression Inventory, Second Edition (BDI-II) [18]: a 21-item self-report that provides a total score index of current depression symptom severity. Each item of the BDI-II is scored from 0 to 3, for a total of 0-63. Higher values represent worse outcomes.
- The World Health Organization Quality of Life scale [19]: a 26-item clinician-administered structured interview that assesses psychological functioning and quality of life in four primary domains: social relationships, psychological, physical, and environment. Each of the 26 questions is scored from 1 to 5, and for each of the 4 domains, a total raw score and 2 transformed scores (with ranges of 4-20 and 0-100) are derived. Higher values represent better outcomes.
- Brief Medication Questionnaire [20]: a self-report of medication use, including what medications the participant was currently taking, how they took each medication in the past week, drug effects and bothersome features, and difficulties remembering to take their medication.

#### Safety

Safety assessments consisted of collecting all adverse events (AEs), serious AEs (SAEs), vital signs, and the InterSePT Scale for Suicidal Thinking–Plus [21,22]. The InterSePT Scale for Suicidal Thinking–Plus is a semistructured interview that assesses the severity of suicidal ideation and behavior, consisting of three parts. Part 1 collects information on 7 days before the visit; there are 13 items that scored 0 (minimum) to 2 (maximum) for suicidality, with a higher score representing a worse outcome. Part 2 collects information on suicidal behavior from the last visit, with nominal categories yes, no, or unknown. Part 3 provides a global rating of status at the time of interview; it is scored 0 (minimum) to 5 (maximum) for suicidality, with a higher score representing a worse outcome. In our study, we focused primarily on the part 3 score (severity of suicidal risk), which was summarized by treatment group and time visit.

#### Other Assessments

Clinician-reported outcomes included the Clinical Global Impression (CGI) scale [23] and the Clinician Satisfaction Survey assessing the clinician’s experience with PEAR-004 and the associated web portal. The CGI consists of the CGI-Severity scale, scored 1 to 7, with larger values indicating greater severity of illness, and the CGI-Improvement scale, scored 1 to 7, with smaller values representing a greater degree of improvement (1=very much improved) and larger values representing a greater degree of worsening (7=very much worse).

Patient-reported outcomes included the Insomnia Severity Index (ISI) [24] and a Subject Satisfaction Survey assessing the participant’s experience with PEAR-004 or sham. The ISI includes 7 questions, each scored from 0 to 4, for a total of 0 to 28, where higher values represent more severe insomnia.

### Statistical Methods

#### Sample Size and Power

The required sample size was calculated to address the primary objective of treatment comparison at week 12 with respect to the change in the total PANSS score. Data from 102 participants randomized in a 1:1 ratio to PEAR-004 or sham control would provide 80% power to detect a statistically significant difference between the 2 groups at a 1-sided significance level of 5% assuming the true standardized effect size of 0.5, which is considered a moderate effect size. To account for potential dropouts, 112 participants were enrolled and randomized into the study.

#### Statistical Analyses

Summary statistics were tabulated for demographics, baseline characteristics, relevant medical histories, and current medical conditions at baseline. The measures of patient engagement derived from the app were explored graphically and using descriptive statistics.

The primary efficacy end point, change in the total PANSS score from baseline to day 85 or last visit, was analyzed using the mixed effects model for repeated measures (MMRM) [25], applied on the intention-to-treat (ITT) set, which included all participants to whom study treatment was assigned by randomization and who had a baseline observation and at least one postrandomization observation for the analysis end point. The MMRM included fixed, categorical effects of treatment, visit, and treatment-by-visit interaction, as well as the continuous, fixed covariates of baseline score, baseline score–by–visit interaction, and disease duration at baseline. An unstructured covariance structure was used to model the within-patient errors. The Kenward-Roger method was used to adjust the estimated covariance of the mean difference and df. The primary comparison was the treatment contrast on day 85.

The secondary efficacy end points, including positive PANSS score, general psychopathology PANSS score, negative PANSS score, total MAP-SR score, total BDI-II score, and World Health Organization Quality of Life total scores for the four domains (social relationships, psychological, physical, and environment), were analyzed similarly to the primary end point (MMRM on change from baseline values, using the ITT set).

The retention to study treatment was assessed using Kaplan-Meier plots of time to drop out from any cause, on the *all randomized* set. Safety data and additional clinical outcome assessments were analyzed using descriptive statistics.

### Ethics and Informed Consent

The study protocol was approved by the Copernicus Group Independent Review Board (study number 1251398). The study was conducted according to the International Conference on Harmonization E6 Guideline for Good Clinical Practice, which has its origin in the Declaration of Helsinki. Informed consent was obtained from each participant in writing at screening before any study-specific procedures were performed. The study was explained to the participant by the investigator or designee, who answered any questions, and written information was also provided.

## Results

### Study Sample

**Figure 2:**
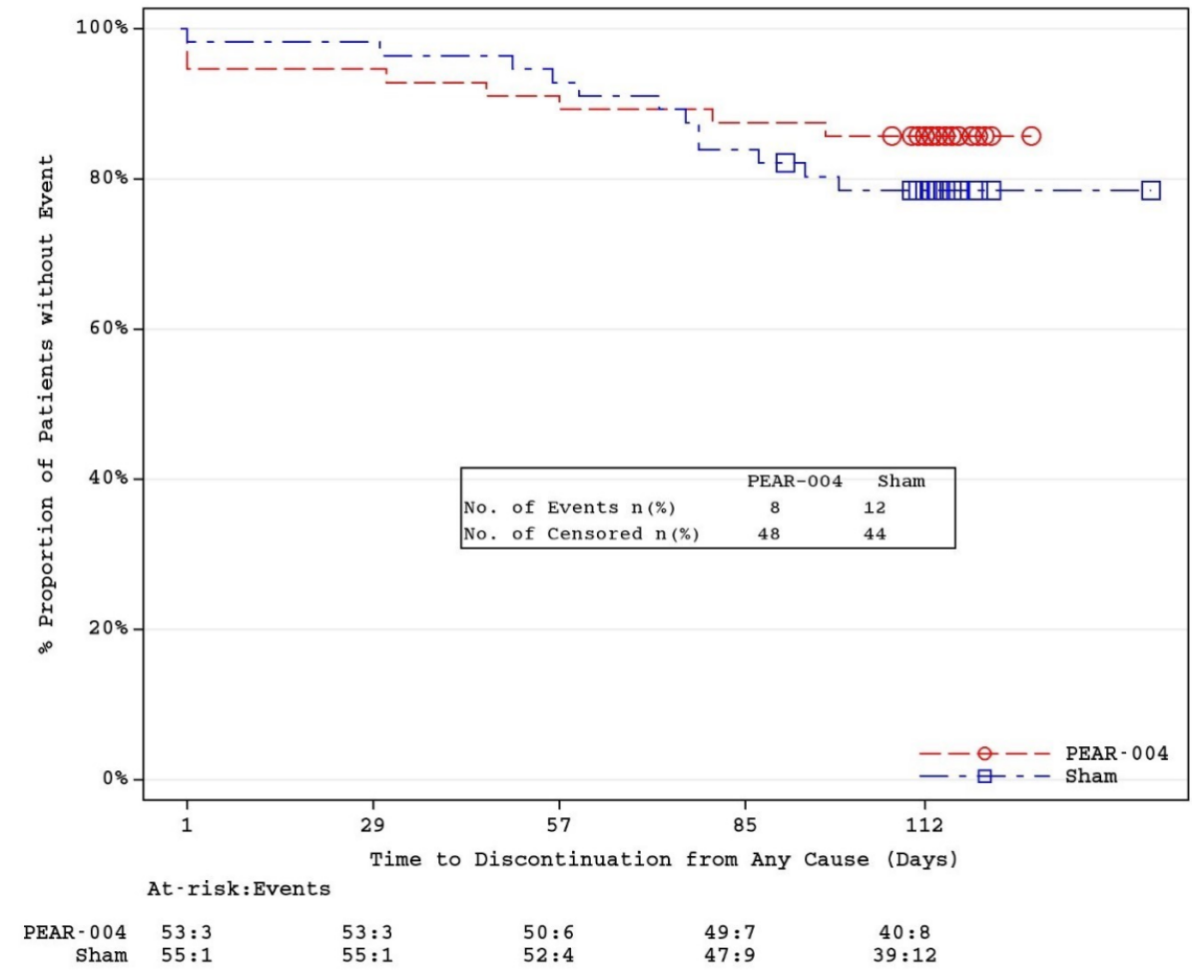
Kaplan-Meier plot of time to dropout (all randomized participants).

From December 10, 2018, to September 26, 2019, a total of 112 participants with schizophrenia were enrolled and randomized into the study (Figure 1). Of the 112 randomized participants, 92 (82.1%) completed the study (48/112, 85.7% from PEAR-004 and 44/112, 78.6% from the sham group). The most common reasons for discontinuation were lost to follow-up and participant or guardian decisions. One participant in the sham group discontinued because of an SAE of suicidal ideation. From the Kaplan-Meier plots (Figure 2), the observed time to discontinuation was somewhat longer in the PEAR-004 group than in the sham group; however, the difference was not statistically significant (log-rank test; P=.36).

### Demographics and Baseline Characteristics

Table 1 describes the demographics and baseline characteristics of study participants. Most participants were men (72/110, 65.5%) and Black or African American (53/110, 48.2%). The mean age of the participants was 45 (SD 11; range 22-65) years. Overall, the treatment groups were similar with respect to background disease characteristics. The most commonly rated participant’s global severity was *moderately ill* (rating 4) in each treatment group (as rated on the CGI-Severity scale at baseline). The mean and median PANSS scores were comparable between the 2 randomized groups. The overall mean duration of disease before study entry was 17.2 (SD 11.5) years, which was balanced across the treatment groups.

**Table 1:** Participant demographics and baseline characteristics (safety analysis set).

Characteristic		PEAR-004 (n=55)	Sham (n=55)	Total (N=110)
**Age (years)**				
	Value, mean (SD)	43.7 (10.99)	45.7 (11.60)	44.7 (11.29)
	Value, median (range)	44.0 (24-64)	48.0 (22-65)	45.5 (22-65)
**Sex, n (%)**				
	Male	36 (65.5)	36 (65.5)	72 (65.5)
	Female	19 (34.5)	19 (34.5)	38 (34.5)
**Race, n (%)**				
	American Indian or Alaska Native	3 (5.5)	0 (0)	3 (2.7)
	Asian	3 (5.5)	4 (7.3)	7 (6.4)
	Black or African American	28 (50.9)	25 (45.5)	53 (48.2)
	Native Hawaiian or Other Pacific Islander	0 (0)	1 (1.8)	1 (0.9)
	White	18 (32.7)	25 (45.5)	43 (39.1)
	Other	3 (5.5)	0 (0)	3 (2.7)
**Total PANSS^a^ score at baseline**				
	Value, mean (SD)	73.5 (10.25)	72.7 (10.10)	73.1 (10.14)
	Value, median (range)	72.0 (61-104)	71.0 (59-106)	72.0 (59-106)
**CGI^b^ severity at baseline, n (%)**				
	3	11 (20)	11 (20)	22 (20)
	4	39 (70.9)	37 (67.3)	76 (69.1)
	5	4 (7.3)	7 (12.7)	11 (10)
	Missing	1 (1.8)	0 (0)	1 (0.9)
**Disease duration at baseline (years)**				
	Value, mean (SD)	16.2 (11.40)	18.2 (11.56)	17.2 (11.47)
	Value, median (range)	13.0 (1-46)	16.0 (1-47)	14.0 (1-47)

^a^PANSS: Positive and Negative Syndrome Scale.

^b^CGI: Clinical Global Impression.

Most participants were taking prior antipsychotic medication at the start of the study. Benzatropine, aripiprazole, quetiapine, and psychiatric medications were the most common medications used for the treatment of schizophrenia in both groups.

### Engagement With App

Table 2 presents a summary of key engagement metrics by the treatment group. Participants in the PEAR-004 group spent significantly more time using the app over the course of the trial than participants in the sham group. However, the sham provided a good control for attention, as there were no significant differences between the groups in the number of days using the app, the total number of sessions, or the number of sessions per day.

**Table 2:** Engagement metrics for the PEAR-004 and sham groups.

Variable		PEAR-004 (n=55)	Sham (n=55)	P value
**Time (hours/day; weeks 1-12)**				
	Value, mean (SD)	4.2 (3.4)	2.2 (4.6)	<.001
	Value, median (range)	3.4 (0-14)	0.8 (0-24)	N/A^a^
**Number of days active**				
	Value, mean (SD)	62.1 (25.8)	63.7 (23.7)	.97
	Value, median (range)	76 (3-87)	71 (1-86)	N/A
**Number of sessions**				
	Value, mean (SD)	257 (160)	320 (295)	.36
	Value, median (range)	241 (9-793)	278 (7-1805)	N/A
**Number of sessions per day**				
	Value, mean (SD)	4.1 (1.7)	4.8 (3.2)	.15
	Value, median (range)	3.6 (2-9)	3.9 (2-21)	N/A

^a^N/A: not applicable.

A more in-depth analysis of engagement data is provided in Tables S1 and S2 in Multimedia Appendix 1. Pear Therapeutics performed poststudy interviews with coordinators at all 6 study sites and found that there were differences among the sites’ onboarding processes and directions on the product use at baseline and throughout the study. These differences in execution may have contributed to the statistically significant site differences in engagement measures observed in the PEAR-004 arm but not in the sham arm. Furthermore, we performed a visual exploration of the primary efficacy outcome (change in total PANSS score at day 85) for PEAR-004 and sham groups across 6 study sites (Figure S3 in Multimedia Appendix 1). There was no evidence that any given site had a between-group difference that would be inconsistent with the primary efficacy analysis of the pooled data across the sites (described in the *Efficacy* section). Therefore, site operational heterogeneity was associated with the measures of engagement with PEAR-004; however, this heterogeneity did not correlate with the observed difference in efficacy between the 2 groups.

### Efficacy

**Figure 3:**
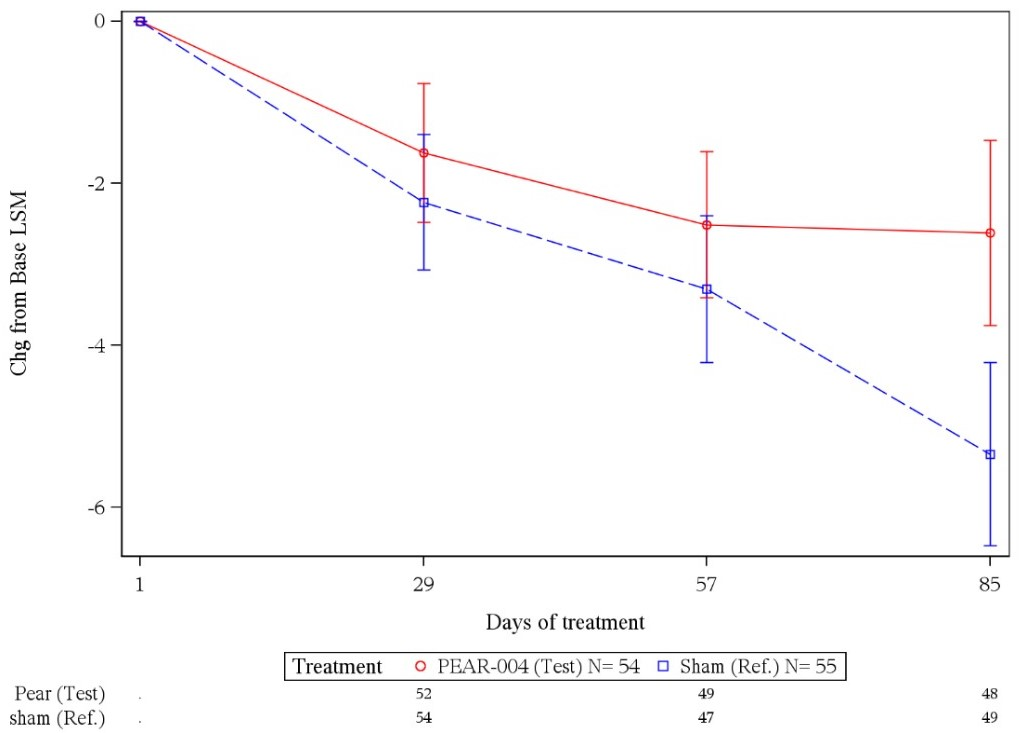
Mixed effects model for repeated measures—estimated mean change (SE) in the total Positive and Negative Syndrome Scale score over time for both groups. Chg: change. LSM: least squares mean. Ref: Reference.

In the primary efficacy end point of change in total PANSS score from baseline to day 85 or the last visit (Figure 3; Table 3), no benefit was seen with PEAR-004 versus sham. The estimated mean change in the PEAR-004 group was −1.6, −2.5, and −2.6 at days 29, 57, and 85, respectively. In the sham group, the estimated mean change was −2.2, −3.3, and −5.3 at days 29, 57, and 85, respectively. Thus, there was a small nondifferential improvement over time in both groups. At day 85, the treatment mean difference between PEAR-004 and the sham group was 2.7 points in favor of the sham (2‑sided P=.09; 90% CI 0.1-5.4).

**Table 3:** Mixed effects model for the repeated measures analysis of change in total Positive and Negative Syndrome Scale scores^a^.

Test vs reference		Value, N			Adjusted least square means (SE)			Comparison of adjusted least square means (test vs reference)	
		Day	Test	Reference	Test	Reference	Difference, test–reference (SE; 90% CI)		P value
**PEAR-004 (n=52) vs sham (n=54)**									
	Analysis 1	29	52	54	−1.6 (0.86)	−2.2 (0.84)	0.61 (1.200; −1.4 to 2.6)		N/A^b^
	Analysis 2	57	49	47	−2.5 (0.9)	−3.3 (0.9)	0.80 (1.279; −1.3 to 2.9)		N/A
	Analysis 3	85	48	49	−2.6 (1.14)	−5.3 (1.13)	2.73 (1.611; 0.1 to 5.4)		.09

^a^Baseline is the last measurement before treatment administration. Model: change from baseline of efficacy end point data was modeled using a mixed effects model with treatment, visit as fixed effects, baseline and disease duration at baseline as continuous covariates, treatment × visit, and baseline × visit interaction effects. The reported P value is 2-sided.

^b^N/A: not applicable.

Table 4 presents the results of secondary efficacy outcomes. No notable benefits were seen except for a small benefit for PEAR-004 in the BDI-II total score at day 57 (least squares mean difference of 3.3 points, PEAR-004 vs sham); however, this difference did not persist at day 85.

**Table 4:** Mixed effects model for the repeated measures analysis of change in secondary efficacy outcome measures^a^.

Variable (PEAR-004 [N=52] vs sham [N=54])			Value, n							Adjusted least square means (SE)					Comparison of adjusted least square means (test vs reference)		
			Day		Test		Reference		Test			Reference		Difference, test–reference (SE; 90% CI)			P value
**Positive PANSS^b^**																	
	Analysis 1	29		52		54		−0.2 (0.36)			−0.4 (0.35)		0.21 (0.501; −0.6 to 1.0)			N/A^c^	
	Analysis 2	57		49		47		−0.8 (0.38)			−1.4 (0.38)		0.61 (0.538; −0.3 to 1.5)			N/A	
	Analysis 3	85		48		49		−1.0 (0.46)			−1.8 (0.45)		0.82 (0.648; −0.3 to 1.9)			.21	
**General psychopathology PANSS**																	
	Analysis 1	29		52		54		−0.8 (0.61)			−1.4 (0.35)		0.51 (0.849; −0.9 to 1.9)			N/A	
	Analysis 2	57		49		47		−1.3 (0.63)			−1.5 (0.38)		0.12 (0.890; −1.4 to 1.6)			N/A	
	Analysis 3	85		48		49		−1.2 (0.76)			−2.8 (0.45)		1.61 (1.071; −0.2 to 3.4)			.14	
**Negative PANSS**																	
	Analysis 1	29		52		54		−0.5 (0.31)			−0.4 (0.30)		−0.13 (0.430; −0.8 to 0.6)			N/A	
	Analysis 2	57		49		47		−0.3 (0.32)			−0.5 (0.32)		0.15 (0.453; −0.6 to 0.9)			N/A	
	Analysis 3	85		48		49		−0.4 (0.41)			−0.9 (0.41)		0.51 (0.581; −0.5 to 1.5)			.38	
**MAP-SR^d^**																	
	Analysis 1	29		51		54		0.8 (1.05)			1.6 (1.02)		−0.79 (1.467; −3.2 to 1.6)			N/A	
	Analysis 2	57		49		47		−0.5 (1.41)			1.1 (1.43)		−1.60 (2.006; −4.9 to 1.7)			N/A	
	Analysis 3	85		48		49		−1.2 (1.26)			2.8 (1.25)		−4.07 (1.780; −7.0 to −1.1)			.02	
**BDI-II^e^ total**																	
	Analysis 1	29		52		54		−1.0 (1.13)			−0.1 (1.11)		−0.92 (−3.6 to 1.7; 1.587)			N/A	
	Analysis 2	57		49		47		−4.8 (1.14)			−1.5 (1.15)		−3.30 (1.629; −6.0 to −0.6)			N/A	
	Analysis 3	85		48		49		−3.4 (1.32)			−3.2 (1.3)		−0.19 (1.855; −3.3 to 2.9)			.92	
**WHOQOL-BREF^f^ domain 1 total**																	
	Analysis 1	29		52		54		−0.1 (0.37)			0.1 (0.36)		−0.18 (0.516; −1.0 to 0.7)			N/A	
	Analysis 2	57		49		47		−0.3 (0.44)			−0.3 (0.45)		0.02 (0.630; −1.0 to 1.1)			N/A	
	Analysis 3	85		48		49		0.2 (0.45)			0.1 (0.45)		0.02 (0.635; −1.0 to 1.1)			.98	
**WHOQOL-BREF domain 2 total**																	
	Analysis 1	29		52		54		−0.6 (0.40)			−0.1 (0.39)		−0.46 (0.562; −1.4 to 0.5)			N/A	
	Analysis 2	57		49		47		−0.7 (0.37)			−0.6 (0.38)		−0.11 (0.528; −1.0 to 0.8)			N/A	
	Analysis 3	85		48		49		−0.5 (0.43)			0.1 (0.42)		−0.57 (0.603; −1.6 to 0.4)			.34	
**WHOQOL-BREF domain 3 total**																	
	Analysis 1	29		52		54		−0.3 (0.28)			0.1 (0.27)		−0.36 (0.389; −1.0 to 0.3)			N/A	
	Analysis 2	57		49		47		−0.3 (0.33)			0.1 (0.33)		−0.33 (0.465; −1.1 to 0.4)			N/A	
	Analysis 3	85		48		49		0.5 (0.33)			0.3 (0.33)		0.19 (0.469; −0.6 to 1.0)			.68	
**WHOQOL-BREF domain 4 total**																	
	Analysis 1	29		52		54		−0.2 (0.61)			−0.9 (0.60)		0.61 (0.853; −0.8 to 2.0)			N/A	
	Analysis 2	57		49		47		−0.5 (0.61)			−0.7 (0.61)		0.25 (0.866; −1.2 to 1.7)			N/A	
	Analysis 3	85		48		49		−0.1 (0.69)			1.1 (0.68)		−1.15 (0.968; −2.8 to 0.5)			.24	

^a^Baseline is the last measurement before treatment administration. Model: change from baseline of efficacy end point data was modeled using a mixed effects model with treatment, visit as fixed effects, baseline and disease duration at baseline as continuous covariates, treatment × visit, and baseline × visit interaction effects. Reported P value is 2-sided.

^b^PANSS: Positive and Negative Syndrome Scale.

^c^N/A: not applicable.

^d^MAP-SR: Motivation and Pleasure-Self Report.

^e^BDI-II: Beck Depression Inventory, Second Edition.

^f^WHOQOL-BREF: World Health Organization Quality of Life.

### Safety

AEs were reported in 20% (22/110) of the participants (Multimedia Appendix 2). The incidence of AEs was similar across both treatment groups: 22% (12/55) for PEAR-004 and 18% (10/55) for the sham group. All reported AEs were categorized as mild (20/110, 18.2%) or moderate (2/110, 1.8%) in severity. No severe AEs were reported. Most AEs were not suspected to be related to the treatment and resolved or were recovering at the end of the study. One SAE (suicidal ideation) was reported in the sham group, and the participant was discontinued from the study. This event was considered resolved on day 43, and was not suspected to be related to the treatment. No clinically significant abnormalities related to any of the vital signs were reported during the study.

### Other Assessments

Table S4 in Multimedia Appendix 1 provides an assessment of the quality of blinding in the study using the sham control app. As shown, the sham app provided only a partial blinding effect, with the direction of bias of unblinding in favor of the PEAR-004 app. As the overall results showed a lack of benefit in the PEAR-004, any bias from partial unblinding would only make any interpretation of hidden potential benefit even more unlikely.

Multimedia Appendix 3 presents a summary of CGI scores at day 85 or the last visit. The proportions of participants with a score of 3 (mildly ill) or 4 (moderately ill) were similar at day 85 in the PEAR-004 group (42/48, 88%) and in the sham group (44/49, 90%). At day 85, 6 participants in the PEAR-004 group and 3 participants in the sham group were markedly ill (score=5). The percentage of participants with a global improvement rating of 4 (ie, no change) was somewhat higher in the PEAR-004 group (29/48, 60%) than in the sham group (23/49, 47%).

There was no change in mean sleep difficulties (ISI score) from baseline to the last visit in the PEAR-004 group (9.5, SD 7.34 vs 9.5, SD 6.82, respectively). A small numerical decrease (ie, improvement) in mean ISI scores was observed in the sham group (baseline: 11.2, SD 7.04; last visit: 9.8, SD 7.37, respectively).

Multimedia Appendix 4 presents a summary of the clinician and patient satisfaction surveys. The Clinician Satisfaction Survey included 4 questions on the clinician’s experience with PEAR-004 (rated 1-7, with 1 indicating a highly negative response and 7 indicating a highly positive response), and the fifth question on how frequently they accessed the web dashboard during the study. Most of the clinicians’ responses had scores 4, showing that the clinicians were satisfied with the PEAR-004 app. The dashboard was accessed at least once per month (21/49, 42% responses), and at least once per week (11/49, 22% responses).

The patient satisfaction survey included 8 questions on the participant’s experience with PEAR-004, each rated from 1 to 7. Most responses to each question received a score of 6 or 7, suggesting that the majority found the app acceptable and usable. Most app use was during mid–late morning, 9 AM to noon (34/49, 69% respondents) and during late afternoon, 3 PM to 6 PM (25/49, 51% respondents). Most frequently, the app was used at home (46/49, 93% respondents) and at a public place (15/49, 30% respondents).

## Discussion

### Principal Findings

The primary objective of this study was to determine whether PEAR-004, a software-based intervention delivered via smartphone, can further reduce symptoms of schizophrenia as measured by the PANSS in participants, almost all of whom are currently on antipsychotic pharmacotherapy. No benefit was seen. Secondary outcomes suggested brief transient improvement in depressive symptoms only. No safety concerns were observed.

The lack of efficacy in this study was not because of a lack of engagement, which was demonstrated to be adequate, or lack of satisfaction. Despite the lack of definable clinical benefit, patients reported that the PEAR-004 app was engaging, interactive, and helped them feel better.

To our knowledge, this study is the largest RCT to date with a sham control group for schizophrenia. Lack of benefit when compared with sham may reflect the nonspecific aspects of benefit seen with digital interventions. It is notable that all participants improved, and if a waitlist control had been used rather than sham, we might have interpreted the results as suggesting mild benefit with the intervention. Natural history of recovery from an acute psychotic exacerbation is also relevant for improvement seen with 12 weeks of follow-up. The inclusion criteria of a moderate psychotic state (PANSS total score ≥60) might have allowed for lower levels of symptomatology, often associated with sham or placebo responses.

This study provides valuable insights that may be useful in future development programs for DTx. The development and selection of a sham control is an important design consideration. In our study, the sham app only included notifications 3 times per day, and when it was opened, it displayed a prescription timer for the remaining duration of app availability. However, this simple control intervention demonstrated a somewhat higher efficacy than PEAR-004. On the basis of feedback from poststudy interviews, the sham app was helpful in focusing attention away from internal stimuli (eg, hearing voices) to the present moment, similar to a mindfulness exercise. The notifications could have been an important ingredient too, as several participants in the poststudy interviews described the receipt of notifications as being meaningful, given their reported lack of social connections. The sham may also present a lower barrier to engagement, which may have been helpful for participants experiencing a greater acuity of clinical symptoms.

### Strengths and Limitations

This study had several strengths. This was a multicenter, randomized, sham-controlled, rater-blinded study design, in which study participants continued on their prescribed clinician-directed pharmacotherapy. Participants randomized to the PEAR‑004 arm had access to the full clinical content and the logic of PEAR-004 app and of its therapeutic components. The sham control arm was chosen to account for the nonspecific effects of engagement with a smartphone. The sham app did not deliver any active coping skills (ingredients of a psychosocial intervention). The analysis of the primary and key secondary efficacy end points was performed on the ITT sample using the MMRM approach, considering all available data from the study participants. The amount of missing data was small, and the results were consistent across the different end points.

There was some heterogeneity in the study implementation aspects by different study sites. From poststudy interviews with coordinators at all 6 sites, we found that there were differences among the sites’ onboarding processes and directions on product use at baseline and throughout the study. A post hoc analysis revealed some evidence of heterogeneity of engagement measures across study sites, which may be linked to a lack of consistency in how study interventions were delivered to participants (Multimedia Appendix 1). However, site operational heterogeneity did not correlate with the difference in efficacy between the 2 groups; in other words, there was no evidence that any site exhibited between-group differences that would be inconsistent with the primary efficacy analysis of the pooled data across the sites. A conclusion from this experience is that it is important to provide detailed instructions to study sites on operational aspects and ensure systematic and similar adherence to those processes.

Furthermore, although PEAR-004 offered clinical content derived from evidence-based treatments such as CBT, in-application support for practicing skills, and applying knowledge to daily life was very limited. This, when combined with the broad therapeutic focus on all patients with schizophrenia (instead of targeting a specific symptom or symptoms), may have inadvertently created an intervention that provided some help to many participants while not adequately supporting the specific needs of any particular participant.

There are several potential approaches for improving the content of PEAR-004 to provide a more personalized treatment delivery. First, to ensure adequate engagement with the CBT mechanism of action, the app could be designed with a utility of personalized goal setting and skill recommendations to help users make progress toward their goals. In addition, creating a web-based environment in which patients can receive just-in-time coaching and medical support may be beneficial. Second, foundational research to better understand users’ needs and how they engage with the digital intervention is essential. The app development should be designed iteratively, evaluating both engagement data and clinical outcomes to see whether the skills that are thought to be important for mechanism of action are adequately practiced; if not, the design should be improved and the assumptions should be re-evaluated in view of accrued experimental data. Finally, the primary goal of this study was to assess the effect of a CBT delivered as adjunct therapy through a mobile app. Additional digital ingredients to optimize patient outcomes, such as medication reminders, tracking of adherence to prescribed medication, and symptom tracking, could be implemented. A challenge would be to assess the added value of each ingredient (and possibly their combinations) when interpreting the trial results. Quantifying engagement with different components of the intervention and properly accounting for it in the analysis (eg, through regression modeling) may be worthwhile. A framework for developing and evaluating complex interventions [26] can be useful in guiding the clinical development of theory-based DTx interventions.

### Comparison With Prior Work

Our results contrast with the three currently available RCTs of digital interventions in schizophrenia, of which all report benefits for delusions, hallucinations, or apathy or withdrawal. However, most of those studies (n=150) were computer-based, not smartphone-based [12]. The other two studies were small, with fewer than 45 participants in each study [10,11]. Further, 2 of the 3 studies had no sham control group and used only waitlist control [11,12]. Another important difference is that unlike previous digital interventions in schizophrenia, PEAR-004 was not integrated into clinical care and no personalized coaching was used to direct and support engagement with treatment. Directing and supporting engagement, whether by a clinician or a trained coach, can help tailor the therapeutic experience for a specific patient. As a prescription digital therapeutic, PEAR-004 was designed to be prescribed by a clinician to their patient and integrated into ongoing care. Not providing this support in this study meant that the burden of knowing what will help was placed on the patient, which may have affected how they engaged with the treatment.

It is instructive to compare the therapeutic content of PEAR-004 with that of some previous mobile-based digital health interventions for people with schizophrenia. Similar to FOCUS [13] and Actissist [10] apps, PEAR-004 content was organized into several domains of cognitive or behavioral strategies, and users received 3 daily notifications prompting them to engage with the app. However, the content of PEAR-004 also included some unique features, such as elements of gamification, to explicitly encourage and incentivize skill mastery through repeated skill practice for generalization of practice to daily life and promote lasting change. Such features may be worth exploring in future studies. In addition, the content of PEAR-004 and future DTx could potentially benefit from the inclusion of short-term and long-term goal settings tailored to specific needs of an individual and mechanisms to help the user achieve their goals. For instance, the PRIME intervention [11] had a self-identified goal setting in the cognitive and behavioral domains, and a supportive web-based community of both age-matched peers with schizophrenia spectrum disorders and motivational coaches.

In this study, the duration of treatment with PEAR-004 or sham was 12 weeks, which is consistent with the results of antipsychotic drug trials in schizophrenia to assess short-term benefits. Proof-of-concept studies of other investigational DTx in psychosis (eg, Actissist [10]; PRIME [11]) have also been conducted for 12 weeks in treatment duration. Furthermore, some other DTx products (eg, Food and Drug Administration–cleared reSET for substance use disorder [27] and reSET-O for opioid use disorder [28]) demonstrated evidence of efficacy following a 12-week treatment period. Although the *optimal* duration for a DTx intervention may vary across different indications, the 12-week treatment duration seems reasonable from the standpoint of balancing engagement and benefits of disease self-management. In this study, there was also a final follow-up visit at week 16. These data were examined descriptively, and the results are available upon reasonable request. Overall, no apparent between-group differences were observed with respect to primary or secondary efficacy outcome measures at week 16. As shown in Figure S5 in Multimedia Appendix 1, the changes in the total PANSS score at week 16 were very similar between the 2 groups.

### Conclusions

In the largest-to-date randomized, sham-controlled study of a digital therapeutic in schizophrenia, PEAR-004 did not demonstrate an effect on the primary outcome of total PANSS scores compared with sham. The secondary and exploratory results also did not demonstrate any notable benefits, except for possible temporary improvement in depressive symptoms. Both clinical investigators and study patients provided high satisfaction ratings for the PEAR-004 app. The study provided many useful scientific and operational insights that can be used in further clinical development of PEAR-004 and other investigational DTx.

## Appendix

Multimedia Appendix 1 Engagement with the PEAR-004 app by study site.

Multimedia Appendix 2 Treatment-emergent adverse events by preferred term.

Multimedia Appendix 3 Clinical Global Impression of Improvement at day 85 or last visit.

Multimedia Appendix 4 Summary of the clinician and patient satisfaction surveys.

Multimedia Appendix 5 CONSORT-EHEALTH checklist (V 1.6.1).

## Abbreviations

AE: adverse event
AVATAR: Audio Visual Assisted Therapy Aid for Refractory auditory hallucinations
BDI-II: Beck Depression Inventory, Second Edition
CBT: cognitive behavioral therapy
CGI: Clinical Global Impression
CONSORT: Consolidated Standards of Reporting Trials
DTx: digital therapeutics
IRT: interactive response technology
ISI: Insomnia Severity Index
ITT: intention-to-treat
MAP-SR: Motivation and Pleasure-Self Report
MMRM: mixed effects model for repeated measures
PANSS: Positive and Negative Syndrome Scale
PI: principal investigator
PRIME: Personalized Real-Time Intervention for Motivation Enhancement
RCT: randomized controlled trial
SAE: serious adverse event
