# Supplementary material for: A Smartphone-Based Intervention as an Adjunct to Standard-of-Care Treatment for Schizophrenia: Randomized Controlled Trial
Source: JMIR Form Res. 2022 Mar 28;6(3):e29154. doi: 10.2196/29154 (PMC9002609; doi:10.2196/29154)
Supplement: Multimedia Appendix 1 [file formative_v6i3e29154_app1.docx]

# Multimedia Appendix 1

## Engagement with PEAR-004 app by study site

The numbers of participants by study site are summarized in Table S1 below. Since randomization was not site-stratified, the treatment numbers were not perfectly balanced for each site (e.g. sites 1002 and 1003 had more subjects randomized to sham, whereas sites 1005 and 1008 had more subjects randomized to PEAR-004).

### Table S1 Number of participants by study site

|  | **PEAR-004** | **Sham** | **Total** |
| --- | --- | --- | --- |
| **Site** | n (%) | n (%) | n (%) |
| 1001 | 11 (20.4) | 10 (18.2) | 21 (19.3) |
| 1002 | 17 (31.5) | 24 (43.6) | 41 (37.6) |
| 1003 | 5 (9.3) | 9 (16.4) | 14 (12.8) |
| 1005 | 9 (16.7) | 3 (5.5) | 12 (11.0) |
| 1006 | 7 (13.0) | 7 (12.7) | 14 (12.8) |
| 1008 | 5 (9.3) | 2 (3.6) | 7 (6.4) |
| **Total** | 54 (100.0) | 55 (100.0) | 109 (100.0) |

Table S2 below presents the results of nonparametric analyses comparing median values of various PEAR‑004 engagement measures across the six study sites. The last two columns show the Kruskal-Wallis test statistics and the corresponding p-values. The p‑values < 0.01 are displayed in **bold font**.

### Table S2 Analysis of PEAR-004 engagement measures across study sites


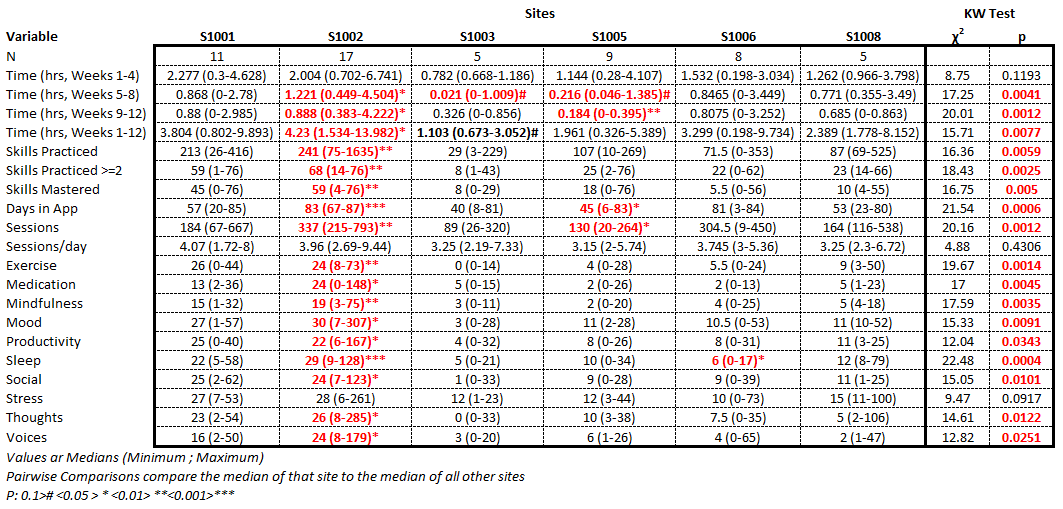


From Table S2, there is evidence of difference among study sites with respect to most of the considered measures. Pairwise comparisons of median values from individual sites vs. median value of all sites revealed some statistically significant results (entries in columns 2–7, displayed in **bold font**). Site 1002 appears to be driving the overall differences.

The observed heterogeneity of engagement measures across study sites may be hypothesized to be linked with lack of consistency in how PEAR-004 was delivered to participants. Notably, there were no site differences in sham engagement (time using the app; data not shown here), which suggests that differences in directing and supporting use uniquely impacted PEAR-004.

Figure S3 below shows box-plot distributions of the primary efficacy outcome (change in total PANSS score at day 85) for PEAR-004 and sham groups across six study sites. Overall, there is no evidence that that any given site had between-group difference that would be inconsistent with the primary efficacy results using pooled data across the sites (cf. Figure 3 and Table 3 in the main paper). Therefore, one can conclude that the site operational heterogeneity was associated with the measures of engagement with PEAR-004; however this heterogeneity did not correlate with the observed difference in efficacy between two groups. As these results are based on relatively low sample sizes per study site, they should be interpreted with caution.

### Figure S3 Box-plots of change in total PANSS score at Day 85 across study sites

## Exploring study implementation aspects

In this study, the directions for use and product onboarding were not explicitly stated in protocol or site operating manual. The reference guide, which was intended to be reviewed with and provided to patients, stated directions for product use in a very general manner (e.g. “...*PEAR-004 works best when you use it regularly and consistently... To get the most out of your time with PEAR-004, you should try to use it every day...*”). The decision to not suggest a specific amount (number of times a day) or type (engaging with a specific feature) of use was so that we could generate the natural proof-of-concept data to understand which patterns of engagement were associated with clinical change. Along these lines, the scripts for the 2, 6, and 10-week calls (appointment reminders from sites to study participants) were structured to not direct or encourage use, but rather to make sure there were no issues preventing participants from using the study product in the way they wanted.

Pear Therapeutics performed post-study interviews with coordinators at all six sites to explore whether the sites had provided consistent onboarding and consistent instructions on the product use to study participants.

There were differences among the sites’ directions on PEAR-004 use at baseline, with only one site following the intention of not directing any amount or type of use. Four of the six sites instructed study participants to use PEAR-004 three times a day (upon notification arrival); however, sites instructed participants to engage with different features. For example, one site reported that they told participants to “*check out a skill if they had time*” while another site “*told them to use the check-in 3 times a day*”.

Similar to PEAR-004, four sites instructed study participants to use sham three times a day, with only one site telling participants to use the sham as much or as little as they wanted. Also similar to PEAR-004, there were differences between sites in their specific instructions, with one site telling participants: “*If you use it on a regular basis or two times a day you can use it as a medication reminder*” and another site telling participants to “*watch the timer for 5-10 seconds and then close*”. For both PEAR-004 and the sham group, one site incentivized a specific amount of use each week by telling participants “*In a week, if they logged 12 or more session, they received $10 for that week.*”

Throughout the study, the sites performed phone call appointment reminders to participants at weeks 2, 6, and 10. The goal was to triage any issues preventing participants from using product as much as they wanted, but not to encourage or direct future use. Only two of the six sites reported that they did not direct use during phone calls. Three sites reported that they directed participants to continue using the study product three times a day during these phone calls (which would be a deviation from the script and protocol). One site continued to incentivize use during these phone calls.

In addition, we included an optional technology screen to help ensure a baseline of digital literacy by requiring participants to have experience with the following: 1) downloading an app on their phone without assistance, and 2) using their phone for making calls, sending text messages, taking pictures, sending emails, and either using the internet, playing games, or using social media. None of the sites reported administering the technology screen, but during interviews site coordinators estimated that approximately 23 out of 112 (21%) participants needed the coordinator to create an email address for them to participate in the study, which would have screened these participants out and speaks to a low baseline digital literacy.

In summary, the described sources of heterogeneity in the sites’ operation may have introduced some extra variability into the study. Some important lessons learned:

- Future studies should ensure a baseline digital literacy.
- Future studies should standardize onboarding to ensure a baseline product knowledge.
- Future studies should standardize directed use at baseline and throughout the study.

## Exploring quality of blinding in the study

At study completion, the participants were asked to provide their feedback (guess) on the treatment they received. Each participant in their response chose one answer from the following options: “PEAR-004”, “Sham”, or “Not sure”. A summary of the results in shown in Table S4 below.

Responses from a total of 85 subjects (40 in the PEAR-004 group, 45 in the sham group) from five study sites were acquired. Overall, 78% of subjects in the PEAR-004 group and 38% of subjects in the sham group guessed their actual treatment correctly. If we define the overall blinding success rate as (number of incorrect or “not sure” responses / total number of responses), the corresponding values are 23% (9/40) for the PEAR-004 group, 62% (28/45) for the sham group, and 44% (37/85) overall in the study.

### Table S4 Assessment of quality of blinding in the study

|  | **Total interviewed** | | **PEAR-004** | | | **Sham** | | |
| --- | --- | --- | --- | --- | --- | --- | --- | --- |
| **Site** | **PEAR‑004** | **Sham** | Guessed correctly | Guessed incorrectly | Not sure | Guessed correctly | Guessed incorrectly | Not sure |
| 1001 | 8 | 10 | 4/8 (50%) | 1/8 (13%) | 3/8 (37%) | 0/10 (0%) | 2/10 (20%) | 8/10 (80%) |
| 1002 | 17 | 20 | 17/17 (100%) | 0/17 (0%) | 0/17 (0%) | 9/20 (45%) | 1/20 (5%) | 10/20 (50%) |
| 1003 | 4 | 6 | 2/4 (50%) | 1/4 (25%) | 1/4 (25%) | 4/6 (67%) | 1/6 (17%) | 1/6 (17%) |
| 1006 | 6 | 7 | 3/6 (50%) | 1/6 (17%) | 2/6 (33%) | 2/7 (28%) | 1/7 (14%) | 4/7 (39%) |
| 1008 | 5 | 2 | 5/5 (100%) | 0/5 (0%) | 0/5 (0%) | 2/2 (100%) | 0/2 (0%) | 0/2 (0%) |
| **Total** | 40 | 45 | 31/40 (78%) | 3/40 (8%) | 6/40 (15%) | 17/45 (38%) | 5/45 (11%) | 23/45 (51%) |

## Exploring efficacy beyond the 12-week treatment period

### Figure S5 Box-plots of change in total PANSS score at days 29, 57, 85, and 115 (End of Study) (weeks 4, 8, 12, and 16)
