# Supplementary material for: A Smartphone-Based Intervention as an Adjunct to Standard-of-Care Treatment for Schizophrenia: Randomized Controlled Trial
Source: JMIR Form Res. 2022 Mar 28;6(3):e29154. doi: 10.2196/29154 (PMC9002609; doi:10.2196/29154)
Supplement: Multimedia Appendix 2 [file formative_v6i3e29154_app2.docx]

# Multimedia Appendix 2

## Treatment emergent adverse events by preferred term

|  | **PEAR-004 (N=55)** | **Sham (N=55)** | **Total (N=110)** |
| --- | --- | --- | --- |
| **Preferred term** | **n (%)** | **n (%)** | **n (%)** |
| Number of subjects with at least one AE | 12 (21.8) | 10 (18.2) | 22 (20.0) |
| Nasopharyngitis | 2 (3.6) | 1 (1.8) | 3 (2.7) |
| Insomnia | 1 (1.8) | 1 (1.8) | 2 (1.8) |
| Upper respiratory tract infection | 1 (1.8) | 1 (1.8) | 2 (1.8) |
| Akathisia | 0 | 1 (1.8) | 1 (0.9) |
| Anxiety | 1 (1.8) | 0 | 1 (0.9) |
| Asthma | 0 | 1 (1.8) | 1 (0.9) |
| Contusion | 0 | 1 (1.8) | 1 (0.9) |
| Decreased appetite | 1 (1.8) | 0 | 1 (0.9) |
| Fall | 1 (1.8) | 0 | 1 (0.9) |
| Gastroenteritis | 1 (1.8) | 0 | 1 (0.9) |
| Genital lesion | 1 (1.8) | 0 | 1 (0.9) |
| Influenza | 0 | 1 (1.8) | 1 (0.9) |
| Joint injury | 0 | 1 (1.8) | 1 (0.9) |
| Latent tuberculosis | 0 | 1 (1.8) | 1 (0.9) |
| Ligament sprain | 1 (1.8) | 0 | 1 (0.9) |
| Nail infection | 1 (1.8) | 0 | 1 (0.9) |
| Osteoarthritis | 1 (1.8) | 0 | 1 (0.9) |
| Palpitations | 0 | 1 (1.8) | 1 (0.9) |
| Peripheral swelling | 0 | 1 (1.8) | 1 (0.9) |
| Plantar fasciitis | 1 (1.8) | 0 | 1 (0.9) |
| Psychotic disorder | 0 | 1 (1.8) | 1 (0.9) |
| Sterile pyuria | 1 (1.8) | 0 | 1 (0.9) |
| Suicidal ideation | 0 | 1 (1.8) | 1 (0.9) |
| Synovial cyst | 0 | 1 (1.8) | 1 (0.9) |
| Tooth abscess | 1 (1.8) | 0 | 1 (0.9) |
| Umbilical hernia | 1 (1.8) | 0 | 1 (0.9) |
| Weight increased | 1 (1.8) | 0 | 1 (0.9) |
| Preferred terms are sorted in descending frequency of AEs in the All treatments column. A subject with multiple AEs is counted only once in the “ at least one AE ” row. A subject with multiple AEs with the same preferred term is counted only once for that preferred term. | | | |
