# Supplementary material for: A Smartphone-Based Intervention as an Adjunct to Standard-of-Care Treatment for Schizophrenia: Randomized Controlled Trial
Source: JMIR Form Res. 2022 Mar 28;6(3):e29154. doi: 10.2196/29154 (PMC9002609; doi:10.2196/29154)
Supplement: Multimedia Appendix 3 [file formative_v6i3e29154_app3.docx]

# Multimedia Appendix 3

## Clinical global impression of improvement (CGI-I) at Day 85 or last visit

|  | **PEAR-004 (N=55)** | **Sham (N=55)** | **Total (N=110)** |
| --- | --- | --- | --- |
| Severity of Illness |  |  |  |
| 1 = Normal, not at all ill | 0 / 48 (0.0) | 0 / 49 (0.0) | 0 / 97 (0.0) |
| 2 = Borderline mentally ill | 0 / 48 (0.0) | 2 / 49 (4.1) | 2 / 97 (2.1) |
| 3 = Mildly ill | 13 / 48 (27.1) | 10 / 49 (20.4) | 23 / 97 (23.7) |
| 4 = Moderately ill | 29 / 48 (27.1) | 34 / 49 (69.4) | 63 / 97 (64.9) |
| 5 = Markedly ill | 6 / 48 (12.5) | 3 / 49 (6.1) | 9 / 97 (9.3) |
| 6 = Severely ill | 0 / 48 (0.0) | 0 / 49 (0.0) | 0 / 97 (0.0) |
| 7 = Among the most extremely ill patients | 0 / 48 (0.0) | 0 / 49 (0.0) | 0 / 97 (0.0) |
| Global Improvement |  |  |  |
| 1 = Very much improved | 0 / 48 (0.0) | 0 / 48 (0.0) | 0 / 97 (0.0) |
| 2 = Much improved | 2 / 48 (4.2) | 2 / 49 (4.1) | 4 / 97 (4.1) |
| 3 = Minimally improved | 12 / 48 (25.0) | 18 / 49 (36.7) | 30 / 97 (30.9) |
| 4 = No change | 29 / 48 (60.4) | 23 / 49 (46.9) | 52 / 97 (53.6) |
| 5 = Minimally worse | 4 / 48 (8.3) | 6 / 49 (12.2) | 10 / 97 (10.3) |
| 6 = Much worse | 1 / 48 (2.1) | 0 / 49 (0.0) | 1 / 97 (1.0) |
| 7 = Very much worse | 0 / 48 (0.0) | 0 / 49 (0.0) | 0 / 97 (0.0) |

**Displayed values are m / n (%), where n = total number of subjects and m = number of subjects with a value for a specific category. Missing values were not replaced, observed cases were used.**
