# Supplementary material for: A Smartphone-Based Intervention as an Adjunct to Standard-of-Care Treatment for Schizophrenia: Randomized Controlled Trial
Source: JMIR Form Res. 2022 Mar 28;6(3):e29154. doi: 10.2196/29154 (PMC9002609; doi:10.2196/29154)

# Multimedia Appendix 4

## Summary of Clinician Satisfaction Survey (N=49)

|  | Response | | | | | | |
| --- | --- | --- | --- | --- | --- | --- | --- |
|  | 1 | 2 | 3 | 4 | 5 | 6 | 7 |
| Q1 | 0 (0) | 1 (2.0) | 1 (2.0) | 14 (28.6) | 16 (32.7) | 14 (28.6) | 3 (6.1) |
| Q2 | 0 (0) | 0 (0) | 1 (2.0) | 22 (44.9) | 6 (12.2) | 17 (34.7) | 3 (6.1) |
| Q3 | 1 (2.0) | 8 (16.3) | 1 (2.0) | 12 (24.5) | 12 (24.5) | 12 (24.5) | 3 (6.1) |
| Q4 | 0 (0) | 5 (10.2) | 3 (6.1) | 19 (38.8) | 8 (16.3) | 13 (26.5) | 1 (2.0) |
|  |  |  |  |  |  |  |  |
|  | a | b | c | d | e |  |  |
| Q5 | 14 (28.6) | 3 (6.1) | 21 (42.9) | 11 (22.4) | 0 (0) |  |  |

Q1: Overall, how satisfied were you with PEAR-004’s impact on the study subjects on a scale of 1-7?

Q2: Do you think PEAR-004 would be a useful addition to standard clinical practice on a scale of 1-7?

Q3: Was the online dashboard a useful addition to the conduct of the trial on a scale of 1-7?

Q4: Do you think the dashboard would be a useful addition to standard clinical practice on a scale of 1-7?

(For Q1 – Q4, 1=Highly negative response; 7=Highly positive response)

Q5: How frequently did you access the dashboard during the study?

(a = Did not access; b = Once; c = At least once per month; d = At least once per week; e = At least once per day)

## Summary of Patient Satisfaction Survey (N=49)

|  | Response | | | | | | |
| --- | --- | --- | --- | --- | --- | --- | --- |
|  | 1 | 2 | 3 | 4 | 5 | 6 | 7 |
| Q1 | 0 (0) | 0 (0) | 1 (2.0) | 5 (10.2) | 7 (14.3) | 9 (18.4) | 27 (55.1) |
| Q2 | 0 (0) | 0 (0) | 1 (2.0) | 3 (6.1) | 3 (6.1) | 7 (14.3) | 35 (71.4) |
| Q3 | 0 (0) | 0 (0) | 2 (4.1) | 6 (12.2) | 4 (8.2) | 13 (26.5) | 24 (49.0) |
| Q4 | 0 (0) | 0 (0) | 2 (4.1) | 4 (8.2) | 4 (8.2) | 13 (26.5) | 26 (53.1) |
| Q5 | 0 (0) | 0 (0) | 3 (6.1) | 4 (8.2) | 4 (8.2) | 15 (30.6) | 23 (46.9) |
| Q6 | 1 (2.0) | 0 (0) | 1 (2.0) | 8 (16.3) | 5 (10.2) | 10 (20.4) | 24 (49.0) |
| Q7 | 0 (0) | 0 (0) | 4 (8.2) | 3 (6.1) | 7 (14.3) | 10 (20.4) | 25 (51.0) |
| Q8 | 0 (0) | 0 (0) | 2 (4.1) | 9 (18.4) | 6 (12.2) | 10 (20.4) | 22 (44.9) |

Q1: Overall, how satisfied were you with the app on a scale of 1-7?

Q2: How easy to navigate was the app on a scale of 1-7?

Q3: How engaging was the app on a scale of 1-7?

Q4: How helpful was the content of the app on a scale of 1-7?

Q5: How relevant was the content of the app on a scale of 1-7?

Q6: How likely would you be to use the app in the future on a scale of 1-7?

Q7: How much did you like receiving daily notifications to use the app on a scale of 1-7?

Q8: How well did the app fit into your daily routine on a scale of 1-7?

For Q1 – Q8, 1=Highly negative response; 7=Highly positive response

## Patient Survey – time and place of PEAR-004 use (N=49)


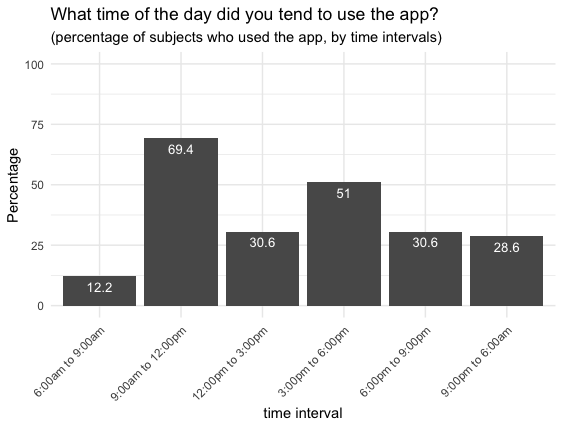


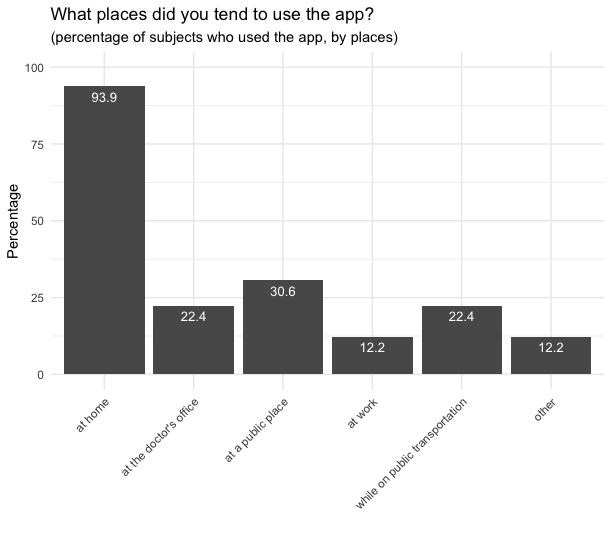

Supplement: Multimedia Appendix 4 [file formative_v6i3e29154_app4.docx]
